# Comparative transcriptomics in *Yersinia pestis*: a global view of environmental modulation of gene expression

**DOI:** 10.1186/1471-2180-7-96

**Published:** 2007-10-29

**Authors:** Yanping Han, Jingfu Qiu, Zhaobiao Guo, He Gao, Yajun Song, Dongsheng Zhou, Ruifu Yang

**Affiliations:** 1 State Key laboratory of Pathogen and Biosecurity, Institute of Microbiology and Epidemiology, Academy of Military Medical Sciences, 20, Dongdajie, Fengtai, Beijing 100071, China

## Abstract

**Background:**

Environmental modulation of gene expression in *Yersinia pestis *is critical for its life style and pathogenesis. Using cDNA microarray technology, we have analyzed the global gene expression of this deadly pathogen when grown under different stress conditions *in vitro*.

**Results:**

To provide us with a comprehensive view of environmental modulation of global gene expression in *Y. pestis*, we have analyzed the gene expression profiles of 25 different stress conditions. Almost all known virulence genes of *Y. pestis *were differentially regulated under multiple environmental perturbations. Clustering enabled us to functionally classify co-expressed genes, including some uncharacterized genes. Collections of operons were predicted from the microarray data, and some of these were confirmed by reverse-transcription polymerase chain reaction (RT-PCR). Several regulatory DNA motifs, probably recognized by the regulatory protein Fur, PurR, or Fnr, were predicted from the clustered genes, and a Fur binding site in the corresponding promoter regions was verified by electrophoretic mobility shift assay (EMSA).

**Conclusion:**

The comparative transcriptomics analysis we present here not only benefits our understanding of the molecular determinants of pathogenesis and cellular regulatory circuits in *Y. pestis*, it also serves as a basis for integrating increasing volumes of microarray data using existing methods.

## Background

*Yersinia pestis *is the etiological agent of plague, alternatively growing in fleas or warm-blood mammals [1]. Fleas acquire this organism via blood meal from a bacteremic mammal, usually a rodent. To produce a transmissible infection, *Y. pestis *colonizes the flea midgut and forms a biofilm in the proventricular valve optimally at 20 to 26°C, blocking its normal blood feeding [2]. Human beings are occasionally infected by directly contacting infected animals or by being bitten by the blocked fleas. Thus, *Y. pestis *must experience a temperature shift during the transmission process between rodents, fleas, and humans. It is considered a facultative intracellular pathogen. After the initial subcutaneous invasion, the bacteria migrate into the regional lymph nodes via the subcutaneous lymph vessel. Most of the organisms that invade the lymph nodes are engulfed and killed by the polymorphonuclear leukocytes (PMNs) that are attracted to invasion sites in large numbers. However, a few bacilli are taken up by tissue macrophages, providing a fastidious and unoccupied niche for *Y. pestis *to synthesize virulence determinants [3]. Residence in this niche also facilitates the bacteria's resistance to phagocytosis [4,5]. The moiety escaped from macrophages can multiply outside of host cells and eventually cause systemic infection. The hypothesized prevailing conditions of phagolysosomal microenvironments include acidic pH, oxidative stress, iron scavenging, nutrition limitation, and killing or inhibiting activities of antibacterial peptides. To survive these stressful environments, *Y. pestis *likely makes appropriate adaptive responses, primarily reflected by the transcriptional changes of specific sets of genes.

A DNA microarray is able to determine simultaneous changes in all the genes of a cell at the mRNA level [6]. We and others have measured the gene expression profiles of *Y. pestis *in response to a variety of stimulating conditions (stimulon analysis), including temperature alteration tolerance [7-9], increased osmolarity [10], ion deficiency [11], antibiotic treatment [12,13], oxidative and acidic stresses [14], antibacterial peptide treatment [14] and nutrition limitation. We also identified the regulons controlled by each of the regulatory proteins, Fur [11], PhoP [15], OmpR, and OxyR, by comparing the gene expression patterns of the mutant transcriptional regulator with that of its parental strain. In order to acquire more regulatory information, all available microarray data of *Y. pestis *including those published signature expression profiles [8-13,15] were collected and subjected to clustering analysis, which infers functionality to the clusters of co-regulated genes.

The transcriptional and genomic information gleaned from coordinately regulated genes was also used to computationally search for potential operons (operon prediction) and *cis*-acting DNA regulatory motifs (motif discovery). Some important findings were further verified by biochemical experiments, including RT-PCR and gel shift assays. This analysis provides an opportunity to gain a global view of environmental modulation of gene expression patterns in *Y. pestis*.

## Results and Discussion

Comprehensive analysis of large sets of microarray expression data is useful to dissect bacterial adaptation to various environments and to understand bacterial gene transcriptional regulation [16,17]. For example, Kendall and his colleagues have compared the general responses of *Mycobacterium tuberculosis *induced by a variety of different *in vitro *conditions (low pH, low nutrients, nitrogen, oxygen stress, stationary phase, and nutrition starvation) [18]. After the determination of the CsrA, SlyA, and PhoPQ regulons in *Samonella typhimurium*, the relevant regulon members are monitored to define the synergetic or antagonistic roles between these three regulators in cell infection models [16].

Recently, many signature expression profiles of *Y. pestis *have been reported [7-13,15,19-21]. All the microarray expression data from our laboratory were analyzed using standardized microarray procedures such that they are suitable for comprehensive analysis. Comparative transcriptomics analysis presented here can be used to mine the regulatory information from these available microarray data, providing an opportunity to gain a global view on environmental modulation of gene expression in *Y. pestis*. This analysis provides an additional dividend towards the transcriptional regulatory networks of *Y. pestis*.

### Virulence genes in response to multiple environmental stresses

In this work, 25 expression profiles of *Y. pestis *were collected for further integration. We hypothesize that the stress conditions used in these experiments will be encountered by this bacterium during its infection and life cycle. The data supported the notion that *Y. pestis *has evolved its ability to coordinately regulate a wide set of genes to survive a wide range of environmental perturbations. Almost all of the known virulence genes were active in the stress responses. Thus, identification of the expression patterns of virulence genes upon a wide set of environmental changes will provide a reference to screen for uncharacterized genes that shown the same differential gene expression under the same stressful conditions.

**Figure 1:**
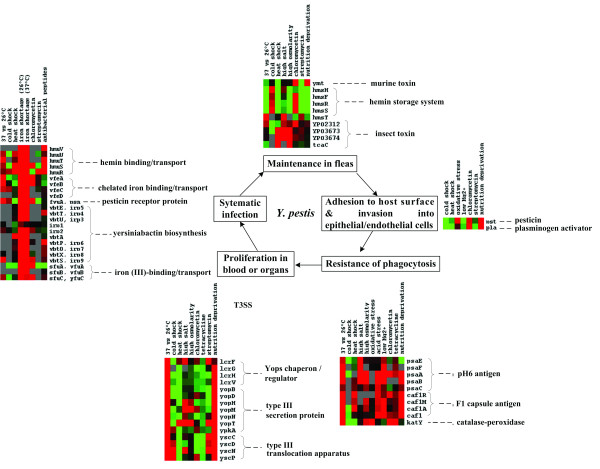
**Environmental modulation of expression of virulence genes**. Shown in the squares are the putative stages of transmission/infection of *Y. pestis*. The TreeView charts show the transcriptional changes of the virulence genes, where columns represent different microarray experiments, and rows represent genes. Color intensities denote log_2 _ratios as follows: green, negative; black, zero; red, positive; gray, missing data.

The transmission and infection of *Y. pestis *can be roughly divided into stages of maintain in fleas, adhesion to host surface, invasion into epithelial or endothelial cells, intracellular growth, antiphagocytosis, and extracellular proliferation (Figure 1). *Y. pestis *possesses a set of virulence determinants that promote infection in mammalian hosts and/or transmission by flea vectors, and different virulence genes have been proven or proposed to be involved in different infection stages (reviewed in [1,22]).

As described previously, expression profiles of *Y. pestis *showed that almost all the putative virulence genes of *Y. pestis *were differentially regulated upon temperature alteration [7-9]. Our data showed that *Y. pestis *known virulence genes also respond to other environmental stresses besides temperature shift (Figure 1). For example, the hemin storage locus, *hmsHFRS *[23], was repressed by temperature upshift, high osmolarity, nutrition limitation, and streptomycin treatment. The *ymt *gene encoding *Yersinia *murine toxin [24] was also regulated by temperature upshift and streptomycin treatment.

*Y. pestis *synthesizes several antiphagocytic factors, including F1 capsular antigen [25], pH6 antigen [4] and *Yersinia *outer proteins (Yops) [26]. Expression of Yops was regulated by temperature alteration, increased osmolarity, and nutrition deficiency under normal Ca^2+ ^condition. These data suggest that the low-calcium response of type III secretion system (T3SS) appears to be triggered at the mRNA level by other environmental cues in addition to temperature upshift and Ca^2+ ^limitation. F1 capsular antigen is expressed much more at 37°C than at 26°C [27]. pH6 antigen (PsaA), encoded by the chromosomal *psaA *gene, expresses *in vitro *between pH 5 and 6.7 at 35 to 41°C [28], or when bacteria live within phagocytic phagolysome [29]. The *psaEFABC *operon encodes a chaperone/usher pathway involved in the secretion and assembly of pH6 antigen as a polymer (fimbriae) on the surface of *Y. pestis *in macrophages [28,30]. PsaE is thought to be a positive regulator of the *psaABC *locus and is required for maximal expression of the pH6 antigen [31]. A recent study showed that the *psaEFABC *locus is regulated by RovA [32]. The microarray data showed that the F1 operon was upregulated upon temperature upshift, low pH medium, oxidative stress, low Mg^2+^, and nutrition deficiency, while the *psaEFABC *locus was induced by temperature alteration, acid stress, low Mg^2+^, nutrition limitation, high salinity and hyperosmotic stress. It is reasonable to assume that synergetic operation of complicated microenvironments within mammalian hosts account for the full expression of these two loci.

### Prediction of operons from microarray data

Operon prediction is the first step toward elucidating gene regulation and reconstructing regulatory networks. Most approaches for prokaryotic operon prediction were developed on the basis of genomic and/or phylogenetic information [33]. For these methods, training with experimental information of known operons is required to generate the predictors. However, little experimental data of operon structure is currently available for *Y. pestis*. To predict the potential operons of *Y. pestis*, we attempted a method that incorporated the empirical correlation coefficient from microarray expression data with the genomic annotation data, including gene orientation, intergenic distance, functional similarity, and intra-genome conservation.

### Stress-responsive operons predicted from microarray expression data

By using the criteria described in Methods, we identified 39 potential operons that consisted of 183 genes in *Y. pestis *(Table 1). Nineteen of these potential operons have been previously studied in other bacteria. There was good agreement between our results and a recent report in which the adjacent genes of *Y. pestis *CO92 are predicted to be within an operon based on the greater conservation of operons in multiple species [33].

**Table 1:** Stress-responsive operons in *Y. pestis *predicted from microarray expression data

**Potential operon (r value)**	**Gene ID**	**Putative or predicted function**	**Reference (s)**
**Iron uptake or heme synthesis**			
*yfeABCD *operon* (r > 0.91)	YPO2439-2442	Transport/binding chelated iron	*yfeABCD *[54]
*hmuRSTUV *operon (r > 0.90)	YPO0279-0283	Transport/binding hemin	*hmuRSTUV *[55]
ysuJIHG* (r > 0.95)	YPO1529-1532	Iron uptake	**-**
sufABCDS* (r > 0.90)	YPO2400-2404	Iron-regulated Fe-S cluster assembly?	**-**
YPO1854-1856* (r > 0.97)	YPO1854-1856	Iron uptake or heme synthesis?	**-**
**Sulfur metabolism**			
*tauABCD *operon (r > 0.90)	YPO0182-0185	Transport/binding taurine	*tauABCD *[56]
*ssuEADCB *operon (r > 0.97)	YPO3623-3627	Sulphur metabolism	*ssu *operon [57]
*cys *operon (r > 0.92)	YPO3010-3015	Cysteine synthesis	**-**
YPO1317-1319 (r > 0.97)	YPO1317-1319	Sulfur metabolism?	**-**
YPO4109-4111 (r > 0.90)	YPO4109-4111	Sulfur metabolism?	**-**
**Urea uptake and urease activation**			
*ure *operon* (r > 0.96)	YPO2665-2672	Pathogenicity	*ure *[58, 59]
**Stress response and adaptation**			
*dnaKJ *operon (r = 0.97)	YPO0468-0469	Chaperones, chaperonins, heat shock	*dnaKJ *[60, 61]
*hslUV *operon (r = 0.97)	YPO0105-0106	Adaptions and atypical conditions	*hslUV *[62]
*katY-cybCB *operon* (r > 0.90)	YPO3319-3321	Detoxification and electron transport	**-**
*psp *operon (r > 0.90)	YPO2349-2351	Adaptions and atypical conditions	*psp *operon [63]
**Ribosome constituents**			
*rps-rpm-rpl *operon (r > 0.90)	YPO0209-0235	Ribosomal protein synthesis and modification	*rps-rpm-rpl *operon [64]
**Energy metabolism**			
*sdh-suc *operon* (r > 0.92)	YPO1109-1116	Tricarboxylic acid cycle	*sdhCDAB *[65]
*cyo *operon (r > 0.94)	YPO3164-3168	Aerobic respiration	*cyoABCDE *[55]
*nap *operon (r > 0.94)	YPO3036-3040	Electron transport	*nap *operon [66]
*atp *operon (r > 0.93)	YPO4120-4128	ATP-proton motive force	*atpIBEFHAGDC *[67]
*ace *operon* (r > 0.90)	YPO3724-3726	Glyoxylate bypass	*aceBAK *[68]
*nuo *operon* (r > 0.92)	YPO2543-2555	Aerobic respiration	*nuo *operon [69]
**Degradation and transport/binding of amino acids**			
*pro *operon* (r > 0.92)	YPO2645-2647	Transport/binding amino acids and amines	*proVWX *[70]
*ast *operon (r > 0.90)	YPO1962-1966	Degradation of amino acids	*astCADBE *[71]
*gln *operon* (r > 0.91)	YPO2512-2514	Transport/binding amino acids and amines	*glnHPQ *[72]
**others**			
YPO1994-1996* (r > 0.98)	YPO1994-1996	Unknown	**-**
YPO0881-0884 (r = 0.99)	YPO0881-0884	Chemotaxis and mobility?	**-**
YPO1087-1088 (r = 0.99)	YPO1087-1088	Phage-related functions and prophage	**-**
YPO0623-0628* (r > 0.94)	YPO0623-0628	Unknown	**-**
*mur *operon (r > 0.95)	YPO0550-0553	Murein sacculus and peptidoglycan	**-**
*idn *operon (r = 0.96)	YPO2539-2540	Degradation of carbon compounds	**-**
*fad *operon* (r = 0.95)	YPO3766-3767	Degradation of small molecule	**-**
*glg *operon (r > 0.90)	YPO3938-3942	Synthesis and modification of cytoplasmic polysaccharides	*glg *operon [73]
YPO3838-3839 (r = 0.92)	YPO3838-3839	Unknown	**-**
YPO0408-0409* (r = 0.97)	YPO0408-0409	Unknown	**-**
YPO1516-1517 (r = 0.90)	YPO1516-1517	Unknown	**-**
YPCD1.15c-1.17c (r > 0.98)	YPCD1.15c-1.17c	Unknown	**-**
*yscGHIJK *operon* (>0.90)	YPCD1.55-1.57	T3SS constituents	**-**
YPPCP1.08c-1.09c (r = 0.97)	YPPCP1.08c-1.09c	Unknown	**-**

'r' represents the correlation coefficient of adjacent genes; '*' represent the defined operon has the similar expression pattern in two other published microarray datasets [7, 21]; '?' inferred functions of uncharacterized genes; '-' means the corresponding operons have not been experimentally validated in other bacteria.

### Verification of predicted operons by RT-PCR

**Figure 2:**
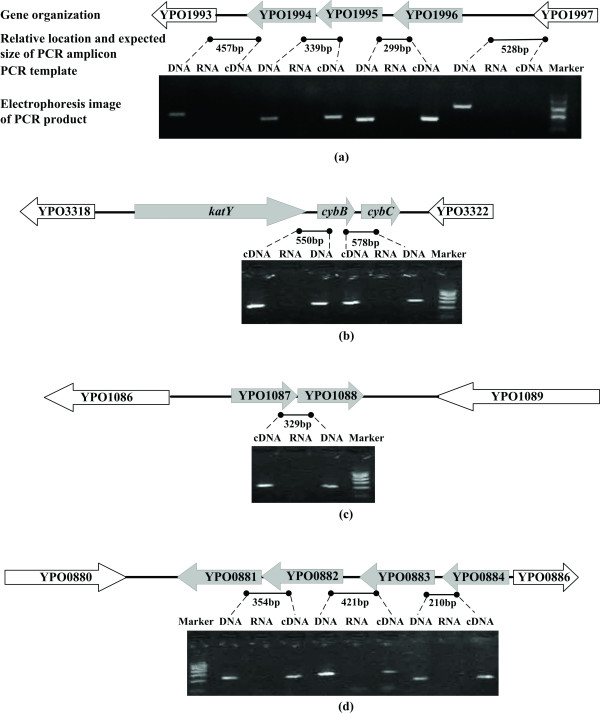
**RT-PCR analysis of potential operons**. Shown is the electrophoresis image of an RT-PCR product with the relative location of the expected size. Total RNA was used to synthesize cDNA in the presence or absence of reverse transcriptase, and the resulting cDNA samples subsequently used for RT-PCR templates, are indicated as "cDNA" or "RNA", respectively. Genomic DNA was used as a template, and is indicated as "DNA" for control PCR. "Marker" represents a DNA size marker (900, 700, 500, 300 and 100 bp from top to bottom).

Four predicted operons were chosen for validation by RT-PCR (Figure 2). Given that genes in an operon are expressed to a single mRNA molecule, reverse transcriptase was used to synthesize first-strand cDNA that was subsequently used as template for PCR. Products were analyzed from the beginning, middle, and end of a multi-gene cluster, so as to define where the multi-gene cluster transcript starts and ends. For the operons, YPO1994-1996 (Figure 2a), *katY*-*cybC*-*cybB *(Figure 2b), and YPO1087-1088 (Figure 2c) analyzed, there was perfect consistency with the above *in silico *prediction. Microarray analysis showed that YPO0881 and YPO0882 were co-expressed, but due to low-quality data it failed to provide the expression data of their downstream genes, YPO0883 and YPO0884. RT-PCR demonstrated that all these four genes were expressed as a single mRNA molecule (Figure 2d), and thus they constituted an operon.

This analysis predicted a total of 39 operons in *Y. pestis*. The advantage of our strategy is that a high accuracy rate would be achieved by integrating microarray experimental data with genomic information. Although there are many bioinformatics tools available for predicting operons, results presented here demonstrated a new reliable strategy for operon prediction and verification, which will be helpful for functional studies. However, only a small number of operons could be predicted, due to the fact i) that only stress-responsive genes were included for this analysis, and ii) that a lot of contiguous gene pairs passed the criteria, but incomplete array data made it difficult to define the border of their primary mRNA transcripts (for example, the extension of a predicted operon, YPO0881-0882 to YPO0881-0884, was demonstrated by RT-PCR as shown in Figure 2d)

### Functional inference of clustering, uncharacterized genes

**Figure 3:**
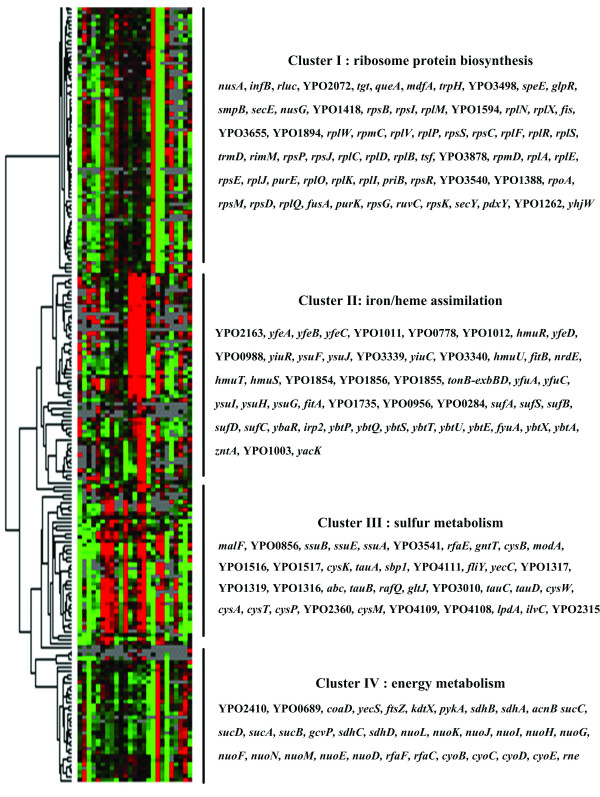
**Schematic representation of the clustered microarray data**. Columns from left to right represent the different microarray experiments from up to down shown in Table 4, while rows from up to down represent genes and their corresponding gene names were listed in the order (left to right and up to down). The black vertical lines are used to define the range of clusters of co-expressed genes. Red represents up-regulation and green represents down-regulation of the corresponding genes.

Clustering microarray expression data can be viewed as a data reduction process, in that observations of gene expression in each cluster can be over-represented (Figure 3). This process provides much greater insight into functional classes of co-expressed genes, since genes that are functionally related should be co-regulated and consequently should show similar expression profiles [34,35]. Thus, clustering genes with similar expression patterns can potentially be utilized to predict the functions of gene products with unknown functions, and to identify sets of genes that are co-expressed and may play the same roles in different cell cycles. We analyzed the expression data with unsupervised algorithms and identified four clusters of co-expressed genes that were associated with ribosome biosynthesis, iron/heme assimilation, and sulfur and energy metabolism. The possible roles of uncharacterized genes may be inferred by referencing other members in each cluster.

### Clustering analysis and functional classification of co-expressed gene clusters

Clustering analysis of the whole microarray dataset was analyzed and four distinct clusters of co-expressed genes, cluster I, II, III, and IV, were identified (Figure 3).

Cluster I consisted of more than 70 genes, most of which are functionally related to biosynthesis of ribosomal proteins. The ribosome is the factory of protein synthesis, and it determines the capacity of the cell to synthesize proteins, thus determining the growth rate of the bacteria. Since most of the members in Cluster I were down-regulated in response to a temperature shift from 26 to 37°C, high osmolarity, Mg^2+ ^limitation, nutrition deficiency, and antibiotics treatment, *Y. pestis *appeared to slow its growth rate under these conditions (see Additional File 1).

Cluster II contains dozens of genes involved in iron/heme assimilation. It is noticeable that almost all of these genes in this cluster were upregulated in response to iron scavenging in wild type (WT) strain, and to iron excess in *fur *mutant grown at 26°C or 37°C. As shown in Table 2, genes in cluster II could be divided into three categories, A (proven), B (putative), and C (hypothetical). Genes in category A (*yfe*, *hmu*, *yfu*, *ybt*, and the *tonB-exbB-exbD *loci) are experimentally proven to be involved in iron/heme assimilation in *Y. pestis *[36-38]. Category B genes showed high degree of similarity with those known to be responsible for iron/heme assimilation in other bacteria. Those genes in category A and B were also found to be iron-responsive in the previously published expression data [11]. However, category C consisted of the *yacK *and *yhhN-zntA *genes that are functionally related to metal metabolism; *sufABCDSE *that encodes constituents of Fe-S cluster assembly [39]; *nrdHIEF *which is responsible for glutaredoxin and ribonucleoside-diphosphate reduction [40]; and some genes (YPO0284, YPO0988, YPO1003, YPO2136, YPO1735, YPO1854-1856, YPO3339) without any functional information. Category C genes are likely indirectly or directly related to iron/heme utilization and metabolism.

**Table 2:** Classification of the gene members of the cluster II in Figure 3

**Gene locus**	**Gene ID**	**Description**	**reference**
**Category A: Proven**			
*yfeABCD*	YPO2439-2442	Inorganic iron and manganese binding/transport system	[36]
*yfuABC*	YPO2958-2960	Inorganic iron transport system	[37]
*ybt *locus	YPO1906-1916	Siderophore-dependent Yersiniabactin biosynthesis and transport	[74]
*hmuRSTUV*	YPO0279-0283	Heme transport system	[38]
*TonB-exbB-exbD*	YPO2193, YPO0682-0683	TonB-ExbB-ExbD complex	[75]
*yiuABCR*	YPO1310-1313	Putative siderophore ABC-transporter	[76]
*ysuFJIHG*	YPO1528-1532	Siderophore biosynthetic enzyme system	[76]
**Category B: Putative**			
*fitABCD*	YPO4022-4025	Putative iron ABC transporter	
Others	YPO0778-0776	putative siderophore biosysnthesis protein	
	YPO1011-1012	putative TonB-dependent outer membrane receptor	
	YPO0956	Putative hydroxamate-type ferri siderophore receptor	
	YPO3340	Putative ferric siderophore receptor (pseudogene)	
**Category C: Hypothetical**			
*sufABCDSE*	YPO2399-2404	Fe-S cluster assembly	
*nrdHIEF*	YPO2648-2651	Ribonucleoside-diphosphate reductase	
*yacK*	YPO3409	Putative exported protein	
*yhhN-zntA*	YPO3819-3820	Zinc, lead, cadmium and mercury transporting ATPase	
Others	YPO0284		
	YPO0988	Putative membrane protein	
	YPO1003	Putative exported protein	
	YPO1735	Putative ABC transporter (ATP-binding protein)	
	YPO1854-1856	Putative membrane or exported protein	
	YPO2163	Putative nitroreductase	
	YPO3339	Hypothetical protein	

Sulfur is one of the nutrients necessary for bacterial life. Genes responsible for sulfur uptake and utilization constitute the *cys *regulon in Gram-negative bacteria [41]. Cluster III contains members of the *cy*s regulon, including *tauABCD*, *ssuEADCB*, *cysPUWAM*, and *sbp1*. These genes were regulated by most of the environmental stresses under study, implying that sulfur metabolism might play important roles in the adaptation of *Y. pestis *to various environmental perturbations. Two genomic loci, YPO1316-1319 and YPO4108-4111, are also included in this sulfur-metabolism-related cluster. Most of the gene products within these two loci were annotated as conserved hypothetical proteins. These two genomic loci might have functions related to sulfur metabolism.

As shown in cluster IV in Figure 3, *sdhCDAB *and *sucABCD *involved in tricarboxylic acid cycle had an expressional pattern similar to that of *nuoA-N *and *cyoABCDE *involved in aerobic respiration. The microarray data showed that these energy metabolism-related genes were down-regulated upon heat shock, high osmolarity, Mg^2+ ^limitation, and streptomycin treatment, but they were upregulated upon chloramphenicol treatment. These results indicated a general retardation of energy generation in *Y. pestis *might occur in response to these suboptimal growth conditions.

### Prediction of regulatory DNA motifs from clustering data

Functionally related members within a cluster of co-expressed genes are likely to be regulated by similar mechanisms; sometimes expression of these genes is even controlled by a single regulatory protein. Promoter DNA sequences containing short (5–20 bp) and relatively conserved regulatory DNA motifs represent the predominant contact sites with the regulatory protein. In this study, the promoter-proximate DNA sequences were collected from each cluster of co-expressed genes. The subsequent motif discovery analysis indicated the presence of DNA motifs that resembled the experimentally proved Fur, PurR, CRP, and Fnr boxes of *E. coli *and other bacteria [42-46], respectively.

### Computational discovery of regulatory DNA motifs

Functionally related members of a cluster of co-expressed genes are likely regulated by similar mechanisms, and even share common *cis*-regulatory DNA elements within their promoter DNA regions. The presence of a motif-like sequence within the upstream region of a gene suggests that it is likely a direct target of the corresponding regulatory protein.

Here, collections of upstream DNA sequences from each of the above four clusters were searched for potential regulatory motifs (Table 3). DNA boxes were found in the promoter regions of each collection. A 16 basepair (bp) box (5'-ACGCAATCGTTTTCNT-3') was detected in the upstream DNA regions of the cluster I genes. It is very similar to the *E. coli *PurR box (5'-ANGMAAACGTTTNCGTK-3') [47]. A 21 bp box (5'-TGATAATGATTATCATTATCA-3') was found for the 19 genes in cluster II. It is a 10-1-10 inverted repeat that resembles the *E. coli *Fur box (5'-GATAATGATAATCATTATC-3') [44]. A 15 bp box (5'-TGANNNNNNTCAA-3') was found within the upstream regions of the cluster III genes. It is a part of the *E. coli *Fnr box (5'-AAWTTGATNWMNATCAAWWWW-3') [45].

**Table 3:** Motif discovery for the clustering genes

**Cluster**	**Genes or operons for motif discovery**	**Strict consensus of known TF-like box (See also Figure 4)**	**Hits of consensus**
Cluster I	*rps-rpm-rpl *operon, *rpsLG*, *rpsF-priB-rpsR-rplI*, *purEK, ruvCAB*, *rpsB*, *rplMI*, *rpsP-rimM-trmD-rplS*, *nusA*-*infB *and *rluC*	PurR-like box: 5' ACGCAATCGTTTTCNT 3'	*rps-rpm-rpl *operon, *purEK, ruvCAB*, *rpsB*, *rpsP-rimM-trmD-rplS*, *nusA*-*infB *and *rluC*
Cluster II	*hmuRSTUV*, YPO0682, YPO0778, YPO0988, YPO1003, YPO1011, *ysuFJIHG*, YPO1735, YPO1854-YPO1856, *irp2-irp1-ybtUTE*, *ybtPQXS*, YPO2163, *sufABCDSE*, *yfeABCD*, *nrdHIEF*, *yfuABC*, YPO3086, YPO3339, *yacK*, *yhhN-zntA *and YPO4022	Fur-like box: 5' TGATAATGATTATCATTATCA 3'	*hmuRSTUV*, YPO0682, YPO0988, YPO1011, *ysuFJIHG*, YPO1735, YPO1854-YPO1856, *irp2-irp1-ybtUTE*, YPO2163, *sufABCDSE*, *yfeABCD*, *nrdHIEF*, *yfuABC*, YPO3086, YPO3339, *yacK*, *yhhN-zntA *and YPO4022
Cluster III	*cysB*, *ssuEADCB*, *cysK*, YPO3541, YPO1517-YPO1516, YPO1316, YPO1317-YPO1319, *fliY*, *sbp1*, *tauABCD*, YPO0186, YPO2360, YPO3010, *cysP*, YPO4112, YPO4108, *ilvC*, YPO2315 and *gntT*	Fnr-like box: 5' TGAN_6_TCAA 3'	*ssuEADCB*, *cysK*, YPO1517-YPO1516, YPO1317-YPO1319, *fliY*, *sbp1*, *tauABCD *and *gntT*
Cluster IV	*sdhCDAB-sucABCD, nuoA-N, cyoABCDE, purB, pta, kbl-tdh, metG, aceE, cysJIH, acnB, murEFXD*, YPO1523, *gph, trpS, pepD, accBC, mutS, ppc, cydAB, fadBA, fadL, fumA, mdh, oppABCDF, treBC, manX, napFDABC *and *frdABCD*	Fnr/Crp-like box: 5' TGANNNNNNTCA 3' ArcA-like box:5' GTTAATTAATGT 3'	*sdhCDAB-sucABCD, pta, kbl-tdh, gph, pepD, mutS, cydAB, fadBA, fumA, oppABCDF, treBC, manX *and *frdABCD acnB, pepD, mutS, mdh, oppABCDF, manX and frdABCD*

A box sequence (5'-TGAN_6_TCA-3') was strictly present in the promoter regions of 14 genes in cluster IV. It is a part of the binding boxes of CRP [43] and Fnr [45]. Previous DNA-binding studies showed that CRP bound to exactly the same sequence as that recognized by Fnr [42]. The ArcA regulator can recognize a relatively conservative sequence (5'-GTTAATTAA-3') [46]. An ArcA-box-like sequence (5'-GTTAATTAATGT-3') was found in the upstream sequence of 7 genes in cluster IV (Table 3).

**Figure 4:**
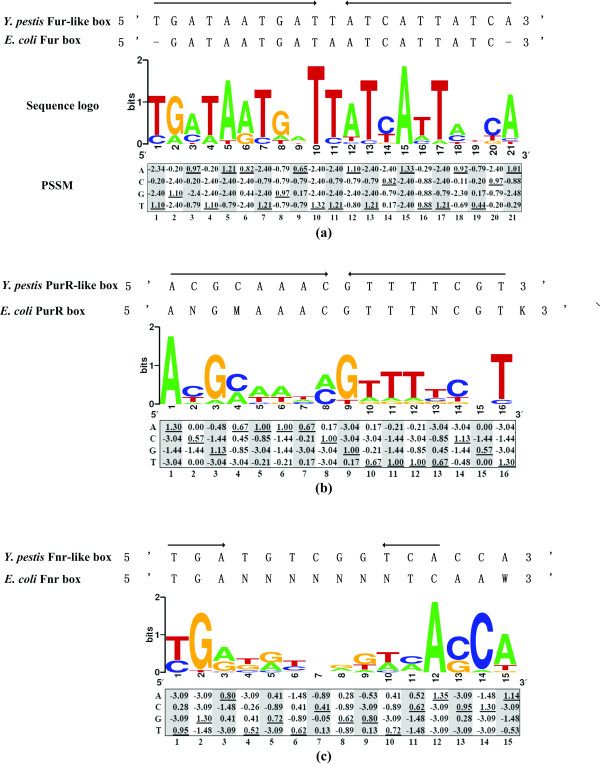
**Graphical representation of the consensus patterns by motif search**. The strict consensus string, sequence logo, and PSSM are included in (a) Fur-like box; (b) PurR-like box; and (c) Fnr-like box. The underlined number is the maximum possible score with PSSM. For the sequence logo, the height of each letter indicates the relative frequency of that base at that position, while the height of each stack of letters corresponds to the sequence conservation at that position.

In addition to the DNA boxes mentioned above that described the regulatory motifs with a contiguous oligonucleotide, we constructed their corresponding position-specific scoring matrix (PSSM; related to a table of probabilistic score of observing nucleotides at each position of aligned sites) (Figure 4).

### EMSA analysis of Fur binding

**Figure 5:**
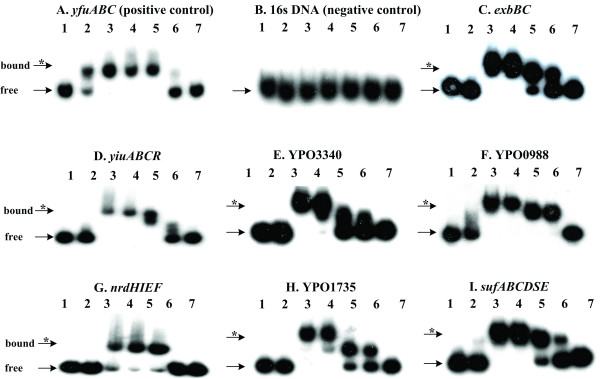
**EMSA analysis of the binding of Fur protein to promoter DNA probes**. Lane 1 contains the rabbit anti-F1 IgG of *Y. pestis*, lane 2 the specific DNA competitor, and lanes 3–7 contain 1.0, 0.7, 0.4, 0.1 and 0 μg of the recombinant Fur protein. In (A) – (I), an arrow and an asterisk indicate the probe (free) and the Fur-probe complex (bound), respectively.

The above motif discovery analysis showed that there were Fur-box-like sequences found in the promoter regions of many genes in cluster II (see Table 3). The presence of a sequence with high similarity with Fur box is a predictor of Fur-specific binding. To validate the motif discovery results, eight genes/operons (*yfuABC*, *exbBD*, *yiuABCR*, YPO3340, YPO0988, *nrdHIEF*, YPO1735, *sufABCDSE*) were chosen from cluster II, covering all three categories shown in Table 2. EMSA (electrophoretic mobility shift assay) was performed to evaluate the binding of Fur to the upstream promoter DNA. Each promoter region was radioactively labeled, incubated with purified His-Fur protein, and then subjected to native gel electrophoresis. The band of free promoter DNA disappears with the increasing amounts of His-Fur protein, and a DNA band with decreased mobility appears, presumably representing the Fur-DNA complex. Thus, the Fur protein binds to the promoter region of each gene/operon tested *in vitro *(Figure 5), indicating that the Fur regulator directly controls the expression of these eight genes/operons.

## Conclusion

The comprehensive transcriptomics analysis benefits our understanding of the molecular determinants of bacterial pathogenesis and cellular regulatory circuits. Our study gave some hints to the possible function of uncharacterized genes and regulatory elements of *Y. pestis *such as operons and DNA regulatory motifs. This kind of analysis would provide an opportunity to gain a global view of environmental modulation of gene expression patterns in *Y. pestis*, which also serve as a basis for integrating increasing volumes of microarray data using existing methods.

## Methods

### Collection of microarray expression data

Table 4 lists all the expression profiles of *Y. pestis *in response to various environmental perturbations. *Y. pestis *strain 201, avirulent to humans, belonging to a newly established biovar, *Microtus *[48], was grown at 26°C in a chemically defined TMH medium [49] to the middle exponential growth phase (A_620 nm _approximately 0.8). Cell cultures were diluted 1:20 in fresh TMH medium, and the cells were grown for at least 10 generations at 26°C until reaching A_620 nm _of approximately 0.8 (26°C cell cultures). If necessary, the cultures were transformed to 37°C for 1 hr (37°C cell cultures). Bacterial cells were then exposed to different *in vitro *stresses (Table 4), and cells were subsequently harvested for RNA isolation. The genome-wide transcriptional changes upon the environmental perturbations were monitored by the standard dual-fluorescent hybridization method with a *Y. pestis *whole-genome DNA microarray spotted with PCR-amplified ORFs [8-10]. For the stimulon analysis, the gene expression pattern of WT strain grown under a stimulating condition was compared with that of an unperturbed control. For the regulon analysis, we compared the expression profiles of deletion mutant strains of the target regulator to WT strain. *Y. pestis *mutants were engineered by replacing the gene targeted for deletion with a Km^r ^encoding cassette via λ-red phage homologous recombination as described previously [11,15].

**Table 4:** Designs for expression profiling of *Y. pestis*

**Environmental perturbation**	**Description**	**Reference (s)**
**Stimulon analysis**		
Temperature shift		[8, 9]
Vegetative growth temperatures	Shift from 26°C to 37°C for 3 hr	
Heat shock	Shift from 37°C to 45°C for 10 min	
Cold shock	Shift from 37°C to 10°C for 1 hr	
Osmotic stress		[10]
High osmolarity	Treatment with 0.5 M sorbitol for 20 min	
High salinity	Treatment with 0.5 M NaCl for 20 min	
Oxidative stress	Treatment with 1 mM H_2_O_2 _for 30 min at 26°C	
Mild acid stress	Shift from pH7.2 to pH 5.5 for 10 min	
Low Mg^2+^	Growth under 10 μM Mg^2+^	[15]
Iron starvation	Treatment with 100 μM of 2, 2'-dipyridyl for 30 min	[11]
26°C	26°C cell cultures	
37°C	37°C cell cultures	
Minimal versus rich medium	TMH versus BHI medium	
Exponential phase	Growth to an A_620 nm _of approximately 0.8	
Stationary phase	Growth to an A_620 nm _of approximately 2.0	
Growth phase	Exponential versus stationary phase	
Minimum medium	Growth in TMH medium	
Rich medium	Growth in BHI medium	
Antibiotics treatment	10× MIC concentration of antibiotics for 30 min	
Streptomycin	37°C cell cultures	[12]
chloramphenicol	37°C cell cultures	
Tetracycline	37°C cell cultures	
Antibacterial peptide	26°C cell cultures	
**Regulon analysis**		
PhoP regulon	*phoP *mutant versus Wide-type (WT) strain	
pH5.5	Growth at pH5.5	
Low Mg^2+^	Growth under 10 μM Mg^2+^	[15]
Fur regulon	*fur *mutant versus WT strain	[11]
26°C	26°C cell cultures with addition of 40 μM of FeCl_3 _for 30 min	
37°C	37°C cell cultures with addition of 40 μM of FeCl_3 _for 30 min	
OxyR regulon	*oxyR *mutant versus WT strain upon exposure to 1 mM H_2_O_2 _for 30 min at 26°C	
OmpR regulon	*ompR *mutant versus WT strain	
High osmolarity	Upon exposure to 0.5 M sorbitol for 20 min	
High salinity	Upon exposure to 0.5 M NaCl for 20 min	

For microarray hybridization, two independent bacterial cultures for each growth condition were prepared as biological replicates for RNA isolation. Three separate labeled probes were made for each RNA preparation as technical replicates. Pairwise comparisons were made using dye swaps to avoid labeling bias.

Whole-genome DNA microarray spotted with 4,005 ORFs was used in our study as described in our previous publications. The data filtering, normalization and significance analysis were done as described previously [9,10]. After filtering the corrupted or suspicious spots during the image analysis phase, expression data of approximately 50%~80% of *Y. pestis *genes on array are available in each array experiment. All the transcriptome data were collected and displayed as a 4,005 × 25 matrix for all the transcriptional changes of 4,005 genes of *Y. pestis *in response to 25 environmental perturbations (see Additional File 2). All the microarray data was deposited in Gene Expression Omnibus (GEO accession number GSE9279).

### Operon prediction

To measure similarities in gene transcriptional regulation, we calculated the correlation coefficient (*r*) of neighboring genes between mRNA transcriptional patterns under multiple environmental conditions. We then further focused our analysis to those genes across all arrays that were differentially regulated in at least five experiments. All the analysis was based on the use of log_2_-ratio of expression values: let *x*_i _and *y*_i _be the log-ratio of fluorescence intensities for a pair of neighboring genes in experiment *i*, where *n *is the number of experiments; *n *= 25. For each pair of genes, we calculated the Pearson correlation coefficient, *r*, between mRNA expression profiles as follows:

r=∑i=1nxiyi−{∑i=1nxi∑i=1nyin}{∑i=1nxi2−(∑i=1nxi)2n}{∑i=1nyi2−(∑i=1nyi)2n}
 MathType@MTEF@5@5@+=feaafiart1ev1aaatCvAUfKttLearuWrP9MDH5MBPbIqV92AaeXatLxBI9gBaebbnrfifHhDYfgasaacH8akY=wiFfYdH8Gipec8Eeeu0xXdbba9frFj0=OqFfea0dXdd9vqai=hGuQ8kuc9pgc9s8qqaq=dirpe0xb9q8qiLsFr0=vr0=vr0dc8meaabaqaciaacaGaaeqabaqabeGadaaakeaacqWGYbGCcqGH9aqpdaWcaaqaamaaqahabaGaemiEaG3aaSbaaSqaaiabdMgaPbqabaGccqWG5bqEdaWgaaWcbaGaemyAaKgabeaaaeaacqWGPbqAcqGH9aqpcqaIXaqmaeaacqWGUbGBa0GaeyyeIuoakiabgkHiTmaacmaabaWaaSaaaeaadaaeWbqaaiabdIha4naaBaaaleaacqWGPbqAaeqaaaqaaiabdMgaPjabg2da9iabigdaXaqaaiabd6gaUbqdcqGHris5aOWaaabCaeaacqWG5bqEdaWgaaWcbaGaemyAaKgabeaaaeaacqWGPbqAcqGH9aqpcqaIXaqmaeaacqWGUbGBa0GaeyyeIuoaaOqaaiabd6gaUbaaaiaawUhacaGL9baaaeaadaGcaaqaamaacmaabaWaaabCaeaacqWG4baEdaqhaaWcbaGaemyAaKgabaGaeGOmaidaaaqaaiabdMgaPjabg2da9iabigdaXaqaaiabd6gaUbqdcqGHris5aOGaeyOeI0YaaSaaaeaadaqadaqaamaaqahabaGaemiEaG3aaSbaaSqaaiabdMgaPbqabaaabaGaemyAaKMaeyypa0JaeGymaedabaGaemOBa4ganiabggHiLdaakiaawIcacaGLPaaadaahaaWcbeqaaiabikdaYaaaaOqaaiabd6gaUbaaaiaawUhacaGL9baadaGadaqaamaaqahabaGaemyEaK3aa0baaSqaaiabdMgaPbqaaiabikdaYaaaaeaacqWGPbqAcqGH9aqpcqaIXaqmaeaacqWGUbGBa0GaeyyeIuoakiabgkHiTmaalaaabaWaaeWaaeaadaaeWbqaaiabdMha5naaBaaaleaacqWGPbqAaeqaaaqaaiabdMgaPjabg2da9iabigdaXaqaaiabd6gaUbqdcqGHris5aaGccaGLOaGaayzkaaWaaWbaaSqabeaacqaIYaGmaaaakeaacqWGUbGBaaaacaGL7bGaayzFaaaaleqaaaaaaaa@8D20@

Genes were assigned to a potential operon if they met the following criterions: i) the correlation coefficient, *r*, of neighboring genes was greater than 0.90; ii) they are transcribed in the same direction and are located on the same strand; iii) intergenic distance between two adjacent genes was shorter than 300 bp and iv) they are functionally linked based on the genomic annotation and/or related publications.

### Verification of predicted operons by RT-PCR

According to the microarray data, genes tested in operon validation were significantly regulated under the growth temperatures of 26°C, 37°C and 45°C. Therefore, RNA was extracted from cells grown at these three temperatures, as done in microarray experiments. Primer pairs were designed for each adjacent gene studied, such that a PCR amplicon across the intergenic region of the two genes would be produced when genomic DNA was used as template (see Additional File 3). cDNA was prepared by reverse transcription with 5 μg of RNA, 200 U of Superscript II Reverse Transcriptase (Invitrogen, Carlsbad, CA) and 3 μg of random hexamer primers. A sample of the resulting cDNA (5 μl) was used for PCR amplification. PCR products were analyzed by agarose gel electrophoresis. To ensure that there was no contamination of genomic DNA, negative controls were performed using RT products with no addition of reverse transcriptase. Reactions containing primer pairs without cDNA template were also included as blank controls.

### Clustering analysis

Genes were clustered according to their expression patterns in the twenty-five different experiments using Cluster 3.0 software [50]. Before clustering, genes with expression data in less than three experiments were removed in order to limit the effects of missing values in the clustering analyses. Of the original 4005 probes, the remaining 3339 probes were used for further clustering analysis. For the original and the filtered data, self-organizing map (SOM) program first ran on the whole microarray dataset to cluster the genes based on the similarity of their expression profiles. The output files were then processed by hierarchical clustering by using the average linkage method. Correlation coefficients of more than 0.6 were arbitrarily extracted and visualized with TreeView [50] to graphically display the data by coloring each cell on the basis of the fluorescence ratio. Cells with log ratios of 0 (no change in gene expression) are colored in black; increasing values of positive and negative log ratios are labeled with increasing intensities of red and green, respectively.

### Discovery of regulatory DNA motifs

For each cluster of co-expressed genes, neighboring genes were grouped into potential operon structures as described above. Promoter-proximate DNA sequences (400 bp upstream to 50 bp downstream of the start codon of each single-gene transcriptional unit, or the first gene of each operon) were retrieved from the genomic sequence of *Y. pesti*s *91001 *with the *retrieve-sequence *tool [51]. Collections of promoter sequences from co-expressed genes within each cluster were searched for potential regulatory DNA motifs with the multiple expectation maximization for motif elicitation (MEME) program [52].

10–30 bases of a single motif and at least 3 distinct motifs in each run were specified. The first two significant (low *E*-value) motifs except AT-rich motifs were chosen for further analysis. The symmetrical or dyad elements were preferentially selected. Then, the sequence logos were built by compilation of the conserved sequences detected in the analysis for each motif with the *WebLogo *program [53]. Finally, PSSM for each DNA motif were built with the *consensus *and convert-matrix tools [51].

### Gel mobility shift analysis of Fur binding

The entire coding region of the *fur *gene of *Y. pestis *strain 201 was cloned into pET28a (Novagen, San Diego, CA). Recombinant plasmids encoding a His-Fur fusion protein were transformed into *Escherichia coli *BL21 (DE3) cells. Expression of His-Fur was induced by addition of isopropyl-β-D-thiogalactoside (IPTG). The recombinant protein was purified under native conditions with a QIAexpressionist™ Ni-NTA affinity chromatography (Qiagen, Valencia, CA). The purified, eluted protein was concentrated to approximately 0.3 mg ml^-1 ^with the Amicon Ultra-15 (Millipore, Billerica, MA). The purity of His-Fur protein was verified by sodium dodecyl sulfate polyacrylamide gel electrophoresis (SDS-PAGE). Primers were designed to amplify the 400–500 bp promoter-proximate region extending upstream from the start codon of each first gene of the operons tested (Table S2 for list of oligonucleotide primers used in this study). EMSA was performed using the Gel Shift Assay Systems (Promega, Madison, WI). The DNA was 5'end-labeled using [γ-^32^P] ATP and T4 polynucleotide kinase. DNA binding was performed in a 10 μl reaction volume containing: binding buffer [100 μM MnCl_2_, 1 mM MgCl_2_, 0.5 mM DTT, 50 mM NaCl, 10 mM Tris-HCl (pH 7.5) and 0.05 mg ml^-1 ^poly-(dI-dC)], labeled DNA (1000–2000 c.p.m. μl^-1^), and increasing amounts of His-Fur protein. We included two control reactions: one contained the specific DNA competitor (unlabeled promoter DNA), while the other was the nonspecific protein competitor [rabbit anti-F1-protein polyclonal antibody (IgG)]. After incubation at room temperature for 30 minutes (min), the products were loaded onto a native 4% (w/v) polyacrylamide gel and electrophoresed in 0.5 × Tris-borate (TB) buffer containing 100 μM MnCl_2 _for 30 min at 220 V. Radioactive species were detected by autoradiography.

## Authors' contributions

YH participated in array experiments, data analysis and manuscript drafting. JQ carried out array experiments and the statistical analysis. ZG and HG carried out RT-PCR and EMSA. YS was involved in experiment design and manuscript revision of this paper. DZ and RY conceived of the study, participated in its design and coordination, and helped to draft the manuscript. Additionally, all authors have already read and approved the final manuscript.

## Supplementary Material

Additional file 1 Figure S1. Growth curves of *Y. pestis *strain 201 under different conditions. Click here for file

Additional file 2 Table S1. All the transcriptional changes of 4005 genes of *Y. pestis *in response to 25 environmental perturbations. Transcriptional changes were presented as the mean log_2 _ratios of mRNA level for each gene under the paired growth conditions (test/reference). The positive number stands for expression level increased, while minus decreased. Row represents the transcriptional pattern of a specific gene under various environmental perturbations and column stands for the genome-wide expression profile in response to a given condition. Click here for file

Additional file 3 Table S2. List of oligonucleotide primers used in this study. Click here for file
