# Supplementary material for: Comparative transcriptomics in Yersinia pestis: a global view of environmental modulation of gene expression
Source: BMC Microbiol. 2007 Oct 29;7:96. doi: 10.1186/1471-2180-7-96 (PMC2231364; doi:10.1186/1471-2180-7-96)
Supplement: Additional file 1 — Figure S1. Growth curves of Y. pestis strain 201 under different conditions. [file 1471-2180-7-96-S1.pdf]

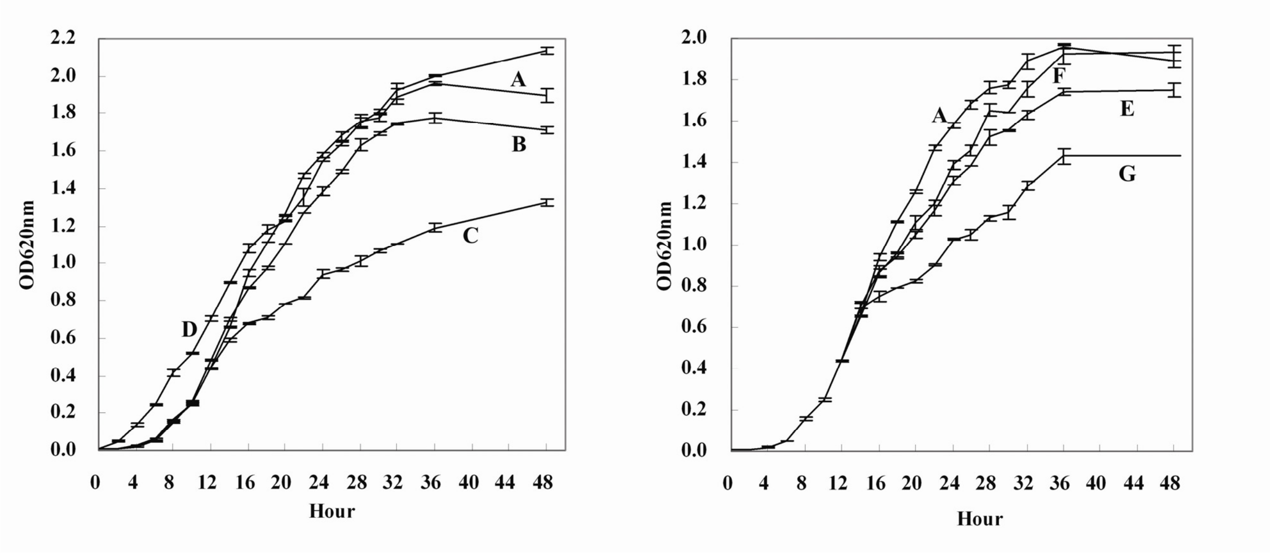

Figure S1. Growth curves of *Y. pestis* strain 201 under different conditions.

Y. pestis strain 201 was grown in TMH at 26°C from 0.001OD to mid-log phase. Cells were pelleted and suspended in 0.9% NaCl-PBS at an OD of 0.8. Cells were then subcultured into TMH in the presence (A) or absence (B) of $CaCl_2$ or TMH with 10μM Mg^{2+} (C) or BHI medium (D) at an OD of 0.01 and grown at 26°C. TMH medium was used for all the experiments but that in rich medium (BHI). Cells in the Ca^{2+}-free TMH grown to 0.2OD was transferred from 26°C to 37°C (B). Cells at an OD of about 0.6 were exposed to 1/10 MIC of antibiotics (E and F) or 0.5M sorbitol (G). At various time points, aliquots were removed from each culture and cell density was monitored with spectrometry.
