# Supplementary material for: Comparative transcriptomics in Yersinia pestis: a global view of environmental modulation of gene expression
Source: BMC Microbiol. 2007 Oct 29;7:96. doi: 10.1186/1471-2180-7-96 (PMC2231364; doi:10.1186/1471-2180-7-96)
Supplement: Additional file 3 — Table S2. List of oligonucleotide primers used in this study. [file 1471-2180-7-96-S3.pdf]

Table S2. List of oligonucleotide primers used in this study

Primer name	Sequence (5’-3')
**For RT-PCR**	
YPO1997F	GCCGATCAGGTATCAAAATTCC
YPO1996R	TACAGGCGTCGTTGAATAAATC
YPO1996F	GGGAAGCGGTCGAAAGC
YPO1995R	GCAACTGAGTGGCAACAC
YPO1995F	TCGCGCCCTGTCTTGG
YPO1994R	AAGGGTTCTCCAACTCGG
YPO1994F	TTCTCACAGTATTGGAAGCATG
YPO1993R	GCATCAGCAATGGCAGAAC
YPO3319F	ACGAATACGCCAGGGCAG
YPO3320R	ATCAAGGGCGGCATTACG
YPO3320F	AATGCCGCCCTTGATGC
YPO3321R	ATTGGTGGTGTCGGGTAC
YPO1087F	CGCTTCGGGGAACTTTGG
YPO1088R	GCTTCTTGCCCTCCTTCC
YPO0882F	GCTGAGGAACTGGGACTG
YPO0881R	ACTCGGCTTCGGTCATG
YPO0883F	CCACCCAATCTGCTGTATCC
YPO0882R	GTTCGGAAACTGCATCACAC
YPO0884F	GAGGGAACACCTGTGATATG
YPO0883R	AGATAAGCTGCTGGTATTCG
**For EMSA**	
YPO2958F	ACAGGGCTATCTGACGGG
YPO2958R	GAAAACGACGTTGCCAGG
YPO0682F	CGTTAGTTCGGCGTGGG
YPO0682R	ACGGGCTGAACCATAGGG
YPO1310F	TGGCGTGACGGCAACC
YPO1310R	AACCCCTTACCTGTTCCC
YPO3340F	AGGGATCACCAGGAAGGG
YPO3340R	ACGCCTCTAGCAAATTTATG
YPO0988F	TTACCGTTGGCTTCTCC
YPO0988R	AACCGTTAATAAAAGTGTGG
YPO2651F	CCTTCCGTGAGTCATTGG
YPO2651R	CCTTCAGGCTGCTATGGG
YPO1735F	GCGTCTTTTATTGGACATGC
YPO1735R	CCGATAAATCAGTTTGAAGC
YPO2404F	GAATGCCTGCGTGAGG
YPO2404R	CACCCGATGAGTTGTTAC
